# Textbook outcome following surgery for pancreatic neuroendocrine tumours: retrospective study

**DOI:** 10.1093/bjsopen/zraf143

**Published:** 2025-12-01

**Authors:** Fabiola A Bechtiger, Zoltan Czigany, Magdalena Lewosinska, Benedict Kinny-Köster, Max Heckler, Ingmar F Rompen, Niels Siegel, Viola Pleines, Maximilian Kryschi, Jörg Kaiser, Mohammed Al-Saeedi, Christoph W Michalski, Markus W Büchler, Martin Loos, Thomas Hank

**Affiliations:** Department of General, Visceral and Transplant Surgery, Heidelberg University Hospital, Heidelberg, Germany; Department of General, Visceral and Transplant Surgery, Heidelberg University Hospital, Heidelberg, Germany; Department of General, Visceral and Transplant Surgery, Heidelberg University Hospital, Heidelberg, Germany; Department of General, Visceral and Transplant Surgery, Heidelberg University Hospital, Heidelberg, Germany; Department of General, Visceral and Transplant Surgery, Heidelberg University Hospital, Heidelberg, Germany; Department of General, Visceral and Transplant Surgery, Heidelberg University Hospital, Heidelberg, Germany; Department of General, Visceral and Transplant Surgery, Heidelberg University Hospital, Heidelberg, Germany; Department of General, Visceral and Transplant Surgery, Heidelberg University Hospital, Heidelberg, Germany; Department of General, Visceral and Transplant Surgery, Heidelberg University Hospital, Heidelberg, Germany; Department of General, Visceral and Transplant Surgery, Heidelberg University Hospital, Heidelberg, Germany; Department of General, Visceral and Transplant Surgery, Heidelberg University Hospital, Heidelberg, Germany; Department of General, Visceral and Transplant Surgery, Heidelberg University Hospital, Heidelberg, Germany; Department of General, Visceral and Transplant Surgery, Heidelberg University Hospital, Heidelberg, Germany; Department of General, Visceral and Transplant Surgery, Heidelberg University Hospital, Heidelberg, Germany; Department of General, Visceral and Transplant Surgery, Heidelberg University Hospital, Heidelberg, Germany

**Keywords:** pNET, pancreas

## Abstract

**Background:**

Recent improvements in pancreatic surgery outcomes have highlighted the relevance of comprehensive quality measures, including textbook outcome. The aim of this study was to evaluate textbook outcome in patients with pancreatic neuroendocrine tumours undergoing surgical resection.

**Methods:**

All patients undergoing surgery for pancreatic neuroendocrine tumours between 2010 and 2023 were included. Textbook outcome was defined as the absence of severe morbidity (Clavien–Dindo grade ≥ III), pancreatic fistula, bile leakage, haemorrhage, readmission, and no death. Logistic regression analysis was used to identify risk factors and Kaplan–Meier survival analysis to compare disease-free and overall survival.

**Results:**

A total of 622 patients underwent surgery for pancreatic neuroendocrine tumours. Major morbidity occurred in 192 patients (30.9%) with an in-hospital mortality rate of 2.6% (16 patients). Rates of postoperative pancreatic fistula, haemorrhage, and readmission were 21.5, 6.4, and 10.3% respectively. Overall, a textbook outcome was achieved in 399 patients (64.1%), with a higher rate after organ-sparing *versus* formal resections (89 (74.8%) *versus* 310 (61.6%); *P* = 0.008). Risk factors for non-textbook outcome were older age (odds ratio 1.52, 95% confidence interval 1.05 to 2.20; *P* = 0.028), higher body mass index (odds ratio 1.61, 95% CI 1.15 to 2.25; *P* = 0.006), American Society of Anesthesiologists grade ≥ III (odds ratio 1.63, 95% CI 1.14 to 2.35; *P* = 0.008), and longer duration of surgery (odds ratio 1.69, 95% CI 1.17 to 2.45; *P* = 0.006). Patients with a textbook outcome had higher 5-year rates of disease-free (73 *versus* 67%; *P* = 0.025) and overall (88 *versus* 78%; *P* < 0.001) survival than those with a non-textbook outcome. This effect was confirmed in patients with non-functioning pancreatic neuroendocrine tumours (overall survival: 85 *versus* 77%; *P* = 0.003). In multivariable analysis, textbook outcome remained an independent predictor of survival.

**Conclusion:**

A textbook outcome was achieved in most patients undergoing pancreatic surgery for pancreatic neuroendocrine tumours and was associated with improved long-term survival. Textbook outcome may serve as a quality control and prognostic indicator in surgery for pancreatic neuroendocrine tumours.

## Teaser

The so-called text-book outcome (TO) has been described as a quality metric for pancreatic surgery. A TO was achieved in 64.1% of patients undergoing surgery for pancreatic neuroendocrine tumours overall, and in 74.8% after organ-sparing resections. Disease-free and overall survival were significantly longer in patients with a TO, suggesting that TO may therefore serve as a prognostic indicator.

## Introduction

Despite significant advances in pancreatic surgery, particularly in minimally invasive techniques, perioperative risks remain high^1–6^. Nevertheless, pancreatic surgery is the only option offering potential cure for localized pancreatic malignancies^7^. Research focused on analysing perioperative complications in pancreatic ductal adenocarcinoma (PDAC)^8,9^ has led to the development of various quality metrics designed to assess surgical outcomes. Recent efforts have aimed at creating standardized tools to compare the qualitative outcomes of pancreatic surgery across different medical facilities^10–14^.

The concept of textbook outcome (TO) encompasses various metrics, such as postoperative pancreatic fistula (POPF) rate, bile leakage, postpancreatectomy haemorrhage, readmission rates, and a Clavien–Dindo classification of ≥ III to define an optimal postoperative scenario. This metric has been applied broadly, with outcomes indicating a significant correlation between achieving a TO and improved postoperative performance^15^. Research has shown that this measure is reliable for comparing results across different centres^16–19^ . Additionally, a surgical–oncological textbook outcome has been considered, which includes complete oncological resection with R0 margins, a minimum of 12 harvested lymph nodes, and appropriate perioperative therapy. A study^12^ has suggested that failing to achieve this outcome correlates with poorer survival rates.

For pancreatic neuroendocrine tumours (pNETs), a study^20^ has also highlighted the importance of TO, associating it with improved disease-free survival (DFS). Recent findings from a study^21^ focusing on non-functioning pNETs has reported mixed results regarding the survival benefits of achieving a TO beyond in-hospital mortality.

The aim of this study was to further investigate TO in surgery for pNETs and its influence on survival rates. This analysis will contribute to understanding of how standardized quality metrics in pancreatic surgery may influence long-term outcomes in this particular patient group.

## Methods

### Patient selection

All patients undergoing surgery for pancreatic neuroendocrine neoplasms from 2010 to 2023 at the University Hospital of Heidelberg were eligible for inclusion. Patients having resection involving all different surgical procedures (parenchyma-sparing and formal resections) were included to represent an all-world population, also including resections of functioning pNETs. Patients undergoing neoadjuvant treatment were also included. All patients underwent preoperative cross-sectional imaging (magnetic resonance tomography or computed tomography) as a part of the staging process. This study was approved by the ethics committee of the Medical Faculty of the University of Heidelberg (S-108/2024) and was performed in accordance with the World Medical Association Declaration of Helsinki as well as the Declaration of Istanbul, and good clinical practice (International conference on harmonization-Good Clinical Practice) guidelines.

### Clinical data collection

Data on patient characteristics, and tumour stage and grade were extracted from the clinic’s digital patient information system. Patient characteristics included height, weight, preoperative treatments, preoperative laboratory values, and American Society of Anesthesiologists (ASA) grade. The type and extent of surgery were analysed, including the number of lymph nodes harvested. Enucleation and segmental resections were summarized as parenchyma-sparing resections. Tumour staging was done according to the eighth tumour node metastasis (TNM) classification of malignant tumours, and grading was based on the Ki-67 (MIB-1) proliferation index on resected specimens^22–24^. Postoperative complications were classified according to Dindo *et al*.^25^. Pancreas-specific complications including clinically relevant POPF, postoperative pancreatic haemorrhage, and delayed gastric emptying were further classified as defined by the International Study Group of Pancreatic Surgery^26–28^. Postoperative bile leakage was graded according to the definition of the International Study Group of Liver Surgery. Readmission was defined as a non-elective readmission within 30 days after discharge. TO was defined by the absence of major complications (grade ≥ III), POPF, bile leakage, postpancreatectomy haemorrhage, 30-day readmission, and in-hospital death^15,16^.

### Oncological follow-up

Comprehensive oncological follow-up was obtained for every patient up to the time of death or last documented contact. Surveillance was conducted either in the institutional specialized outpatient clinic or, for patients who preferred local follow-up, through structured annual telephone interviews. Recurrence was divided into local and systematic recurrence (for example liver, lung, peritoneum). Patients without information on follow-up (such as international patients) were excluded from survival analyses.

### Statistical analysis

Quantitative data are reported as median (interquartile range, i.q.r.). Absolute and relative frequencies were determined for all qualitative variables. Differences in quantitative variables between the two groups were assessed using the Mann–Whitney *U* test, whereas qualitative variables were compared using Fisher’s exact test or the χ^2^ test as appropriate.

Overall survival (OS) was defined as the interval from resection to either death from any cause or the last follow-up, whereas DFS was defined as the interval from resection to the first detection of recurrence or newly diagnosed regional or distant metastases. These survival outcomes were estimated using the Kaplan–Meier method and analysed with the log rank test. Patients who died within 30 days after surgery were excluded. Additionally, patients who were still alive at their last follow-up were censored. Cox regression analysis was used to determine the influence of various factors on survival metrics. All statistical tests were two-sided, with significance set at *P* < 0.050. Data analysis was performed using SAS^®^ version 9.4 (SAS Institute, Cary, NC, USA).

## Results

From January 2010 to November 2023, 622 patients underwent surgical resection for pNETs (*Table 1*). Most patients were men (331, 53.2%) and the median age at the time of surgery was 58.6 (i.q.r. 48.5–67.7) years. The majority were classified as having ASA grade II (400, 64.3%) and the median BMI was 25.8 (i.q.r. 23.0-29.3) kg/m^2^. Most pNETs were non-functioning (537, 86.3%); among functioning tumours, insulinomas were the most common (76, 89.4%). On pathological examination, 278 patients (44.7%) presented with T1 tumours and 329 (52.9%) had no lymph node metastases. The majority of patients had well differentiated tumours (G1: 333, 53.5%) and the R0 resection rate was 72% (Rx: 90, 14.5%). Median follow-up was 49.7 (i.q.r. 19.4–96.1) months for the entire cohort.

**Table 1:** Patient characteristics

	Total (*n* = 622)	TO (*n* = 399)	Non-TO (*n* = 223)	P*
Age (years), median (i.q.r.)	58.6 (48.5–67.7)	57.3 (47.4–66.2)	61.3 (52.1–70.1)	< 0.001†
**Sex**				0.315
Male	331 (53.2%)	206 (51.6%)	125 (56.1%)	
Female	291 (46.8%)	193 (48.4%)	98 (43.9%)	
BMI (kg/m^2^), median (i.q.r.)	25.8 (23.0-29.3)	25.4 (22.6-29.1)	26.4 (24.0-29.4)	0.007†
**ASA grade**				0.022
I	55 (8.8%)	35 (8.8%)	20 (9%)	
II	400 (64.3%)	271 (67.9%)	129 (57.8%)	
III	166 (26.7%)	93 (23.3%)	73 (32.7%)	
IV	1 (0.2%)	0 (0%)	1 (0.4%)	
Functioning tumour	85 (13.7%)	58 (14.5%)	27 (12.1%)	0.465
**Type of surgery**				0.013
Left pancreatectomy	288 (46.3%)	182 (45.8%)	106 (47.5%)	
Pancreatoduodenectomy	159 (25.6%)	100 (25%)	59 (26.5%)	
Total pancreatectomy	56 (9%)	28 (7%)	28 (12.6%)	
Enucleation	109 (17.5%)	83 (20.8%)	26 (11.7%)	
Segmental resection	10 (1.6%)	6 (1.5%)	4 (1.8%)	
Vascular resection	64 (10.3%)	34 (8.5%)	28 (12.6%)	0.125
Minimally invasive approach	124 (19.9%)	83 (20.8%)	41 (18.4%)	0.530
Duration of surgery (minutes), median (i.q.r.)	225 (153–320)	211 (145–300)	247 (182–350)	< 0.001†
**Tumour grade**				0.025
G1	333 (53.5%)	211 (52.9%)	122 (54.7%)	
G2	231 (37.1%)	159 (39.8%)	72 (32.3%)	
G3	57 (9.2%)	28 (7%)	29 (13%)	
**Tumour category**				0.017
T1	278 (44.7%)	184 (46.1%)	94 (42.2%)	
T2	135 (21.7%)	97 (24.3%)	38 (17%)	
T3	200 (32.2%)	114 (28.6%)	86 (38.6%)	
T4	9 (1.4%)	4 (1%)	5 (2.2%)	
**Lymph node status**				0.026
N0	329 (52.9%)	202 (50.6%)	127 (57%)	
N1	174 (28%)	108 (27.1%)	66 (29.6%)	
Nx	119 (19.1%)	89 (22.3%)	30 (13.5%)	
**Distant metastasis category**				0.623
M0	539 (86.7%)	348 (87.2%)	191 (85.7%)	
M1	83 (13.3%)	51 (12.8%)	32 (14.3%)	
**Resection margin**				0.024
R0	448 (72%)	280 (70.2%)	168 (75.3%)	
R1	84 (13.5%)	50 (12.5%)	34 (15.2%)	
Rx	90 (14.5%)	69 (17.3%)	21 (9.4%)	
Length of hospital stay (days), median (i.q.r.)	11 (8–19)	9 (7–12)	23 (13–35)	< 0.001†

Values are *n* (%) unless otherwise stated. TO, textbook outcome; i.q.r., interquartile range; BMI, body mass index; ASA, American Society of Anesthesiologists. *χ^2^ test or Fisher’s exact test, as appropriate, except †Mann–Whitney *U* test.

Most patients underwent left pancreatectomy (288, 46.3%) followed by pancreatoduodenectomy (159, 25.6%), organ-sparing resections (119, 19.1%), and total pancreatectomy (56, 9%). In addition, vascular resections were performed in 64 patients (10.3%). Overall, 192 patients (30.9%) developed major complications with a Clavien–Dindo grade of ≥ IIIa, of which 72 were Clavien–Dindo grade ≥ IIIb (11.6%). POPF was observed in 134 patients (21.5%), whereas 58 (9.3%) experienced a biochemical leak. Postpancreatectomy haemorrhage was noted in 40 patients (6.4%), and 19 (3.1%) had bile leakage. The in-hospital mortality rate was 2.6% and the readmission rate 10.3%.

## Rate and impact of TO

Overall, a TO was achieved in 399 patients (64.1%). Patients who achieved a TO were significantly younger, exhibited lower BMI and ASA grade, had a shorter duration of operation, more favourable pathological findings, and shorter hospital stay (*Table 1*). There was no significant difference in TO rates between functioning and non-functioning pNETs: 58 (68.2%) and 341 (63.5%), respectively (*P* = 0.465).

In analyses stratified by surgical approach, organ-sparing resections had higher TO rates compared with formal resections: 89 (74.8%) *versus* 310 (61.6%) (*P* = 0.008). In the subgroup of formal resections, pylorus-preserving pancreatoduodenectomy had the highest TO rate (64, 67.4%) followed by left pancreatectomy (182, 63.2%), pylorus-resecting pancreatoduodenectomy (36, 56.3%), and total pancreatectomy (28, 50%) (*P* = 0.013).

In univariable logistic regression analysis, older age, BMI > 25 kg/m², ASA grade ≥ III, longer duration of surgery, and total pancreatectomy were associated with a significantly lower likelihood of achieving a TO (*Table 2*). Conversely, early tumour stage (T1/2 *versus* T3/4) was positively associated with TO (odds ratio (OR) 0.61; *P* = 0.005). Minimally invasive surgery, tumour grade, nodal status, and tumour functionality showed no significant association with TO.

**Table 2:** Univariable and multivariable logistic regression analysis for TO

	Non-TO	TO	Univariable analysis*		Multivariable analysis*	
			Odds ratio	*P*	Odds ratio	*P*
Older age (4th quartile)	67 (30%)	88 (22.1%)	1.52 (1.05, 2.20)	0.028	1.42 (0.96, 2.11)	0.083
BMI > 25 kg/m^2^	144 (64.6%)	212 (53.1%)	1.61 (1.15, 2.25)	0.006	1.61 (1.14, 2.27)	0.007
Female sex	98 (43.9%)	193 (48.4%)	0.84 (0.60, 1.16)	0.289		
ASA grade ≥ III	74 (33.2%)	93 (23.3%)	1.63 (1.14, 2.35)	0.008	1.38 (0.94, 2.03)	0.102
Longer duration of surgery (4th quartile)	70 (31.4%)	85 (21.3%)	1.69 (1.17, 2.45)	0.006	1.47 (0.99, 2.18)	0.057
Minimally invasive surgery	41 (18.4%)	83 (20.8%)	0.86 (0.56, 1.30)	0.470		
Total pancreatectomy†	28 (12.6%)	28 (7%)	1.90 (1.10, 3.30)	0.022	1.34 (0.75, 2.42)	0.327
Left pancreatectomy†	103 (46.2%)	182 (45.6%)	1.02 (0.74, 1.42)	0.890		
Pancreatoduodenectomy†	58 (26%)	100 (25.1%)	1.05 (0.72, 1.53)	0.795		
Vascular resection	28 (12.6%)	34 (8.5%)	1.54 (0.91, 2.62)	0.109		
Functioning tumour	27 (12.1%)	58 (14.5%)	0.81 (0.50, 1.33)	0.410		
Tumour category (T1/2 *versus* T3/4)	132 (59.2%)	281 (70.4%)	0.61 (0.43, 0.86)	0.005	0.64 (0.45, 0.92)	0.017
Tumour grade (G1 *versus* G2/G3)	122 (54.7%)	211 (52.9%)	1.07 (0.77, 1.49)	0.685		
Lymph node status N ≥ 1	96 (43%)	197 (49.4%)	0.78 (0.56, 1.08)	0.130		
Metastasis category M1	32 (14.3%)	51 (12.8%)	1.14 (0.71, 1.84)	0.582		
Tumour size ≤ 2 cm	101 (45.3%)	192 (48.1%)	0.89 (0.64, 1.24)	0.498		

Values are *n* (%) unless otherwise stated; *values in parentheses are 95% confidence intervals; †compared to all other types of surgery. TO, textbook outcome; BMI, body mass index; ASA, American Society of Anesthesiologists.

In multivariable logistic regression analysis, BMI > 25 kg/m^2^ (OR 1.61; *P* = 0.007) and advanced T3/T4 tumours (OR 1.55; *P* = 0.017) remained independent predictors of non-TO.

## Survival analysis

**Fig. 1:**
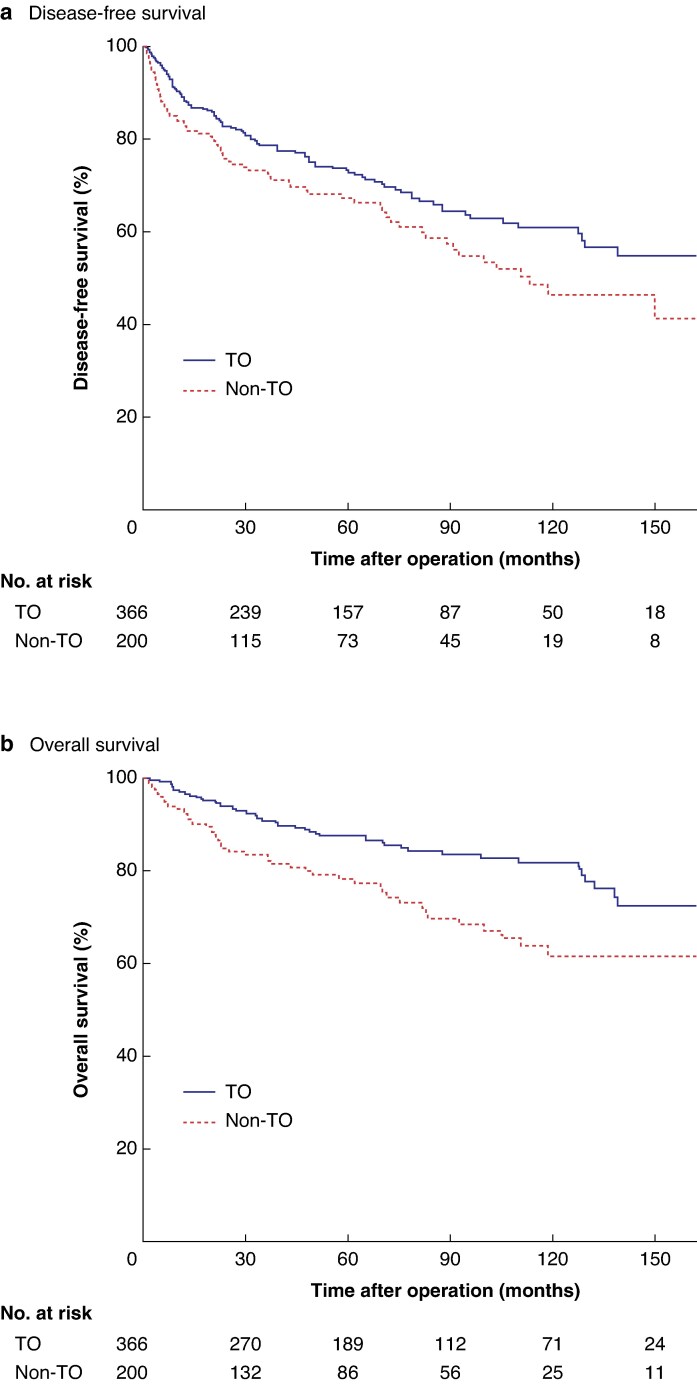
Impact of TO on disease-free and overall survival for all patients, excluding those who died within 30 days after surgery (*n* = 566) **a** Disease-free and **b** overall survival. TO, textbook outcome.

The 5-year DFS rate was 71.1%, with a median DFS of 149.9 months for the entire cohort. Patients who achieved a TO had significantly improved DFS compared with the non-TO group (73.2 *versus* 67.2%; *P* = 0.025). Median DFS was not reached in the TO group, whereas it was 113.25 (95% confidence interval 74.4 to 152.1) months in the non-TO group (*Fig. 1a*). The OS rate of the cohort after 5 years was 84.2%. Patients with a TO had significantly better OS than those without a TO (87.5 *versus* 78.2%; *P* < 0.001); median OS was not reached in either group (*Fig. 1b*).

**Fig. 2:**
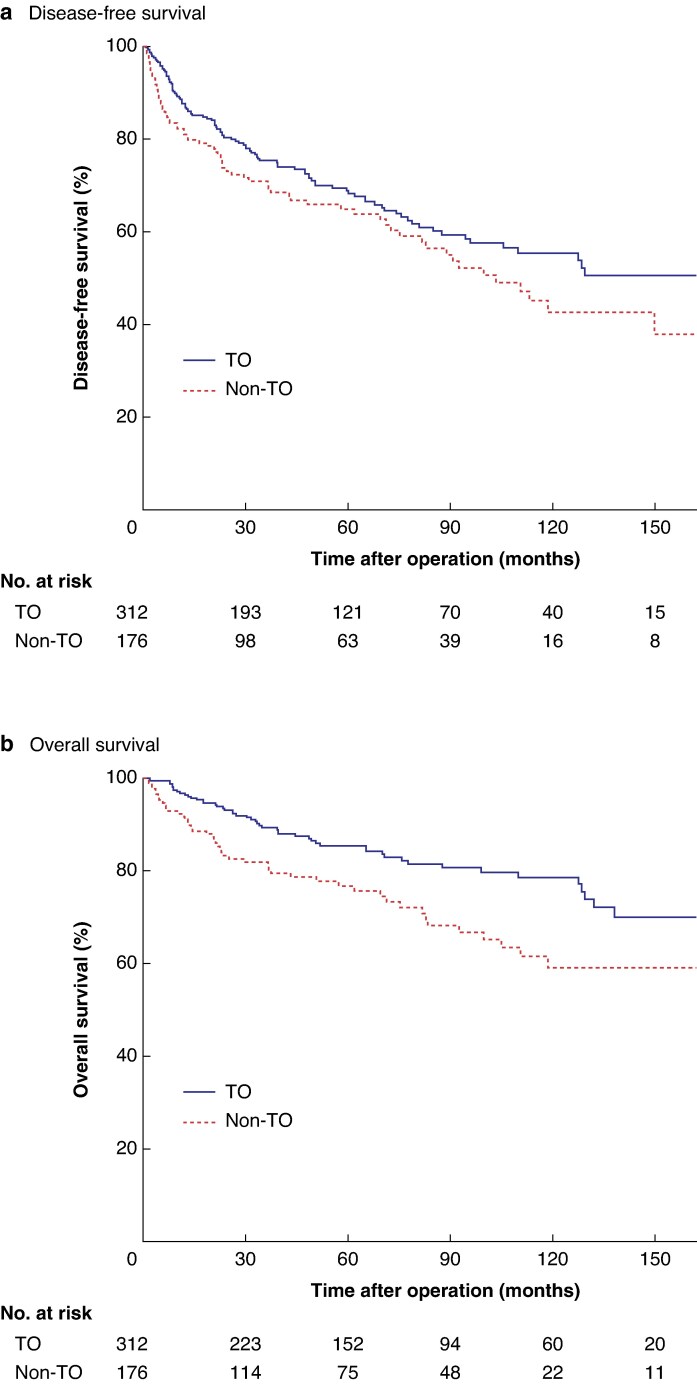
Impact of TO on disease-free and overall survival for patients with non-functioning pancreatic neuroendocrine tumours, excluding those who died within 30 days after surgery (*n* = 488) **a** Disease-free and **b** overall survival. TO, textbook outcome.

In subgroup analysis of patients with non-functioning pNETs, the DFS rate was 68.8% in the TO group *versus* 64.8% in the non-TO group; median DFS was not reached in the TO group whereas it was 103.5 (77.9 to 129.1) months in the non-TO group (*P* = 0.080) (*Fig. 2a*). Similarly, OS was significantly improved in patients with a TO, with a 5-year OS rate of 85.4% compared with 76.7% among those without a TO (*P* = 0.003) (*Fig. 2b*).

In subgroup analysis of patients who had a formal resection, the DFS rate was 67.4% in the TO group *versus* 61.8% in the non-TO group, with a median DFS of 139 and 103.5 months respectively (*P* = 0.096) (*Fig. S1a*). Similarly, OS was significantly improved in patients with TO, with a 5-year OS rate of 83.9% compared with 74.8% in the non-TO group (*P* = 0.012) (*Fig. S1b*).

In multivariable analysis, a non-TO was independently associated with reduced DFS in the entire cohort (hazard ratio (HR) 1.36; *P* = 0.047). In addition, other well established prognostic parameters including older age (HR 1.65; *P* = 0.002), positive lymph node status (HR 1.52; *P* = 0.010), higher tumour grade (HR 2.70; *P* < 0.001), distant metastases (HR 1.99; *P* < 0.001), male sex (HR 1.39; *P* = 0.038), and non-functioning tumours (HR 2.48; *P* = 0.014) were independently associated with reduced DFS (*Table 3*). For OS, non-TO remained a significant independent risk factor (HR 1.68; *P* = 0.011). Additional independent predictors of worse OS were older age (HR 2.90; *P* < 0.001), distant metastases (HR 2.82; *P* < 0.001), and male sex (HR 1.64; *P* = 0.022) (*Table 4*).

**Table 3:** Multivariable Cox regression analysis of disease-free survival

	Hazard ratio	*P*
Older age (fourth quartile)	1.65 (1.20, 2.27)	0.002
Female sex	0.72 (0.52, 0.98)	0.038
Functioning tumour	0.40 (0.20, 0.83)	0.014
Tumour category (T1/2 *versus* T3/4)	0.76 (0.52, 1.13)	0.171
Tumour size ≤ 2 cm	0.66 (0.42, 1.05)	0.079
Lymph node status ≥ N1	1.52 (1.11, 2.08)	0.010
Metastasis category M1	1.99(1.38, 2.86)	< 0.001
Tumour grade (G1 *versus* G2/G3)	0.37 (0.25, 0.55)	< 0.001
Textbook outcome	0.73 (0.54, 1.00)	0.047

Values in parentheses are 95% confidence intervals.

**Table 4:** Multivariable Cox regression analysis of overall survival

	Hazard ratio	*P*
Older age (4th quartile)	2.90 (1.93, 4.34)	< 0.001
Female sex	0.61 (0.40, 0.93)	0.022
Functioning tumour	0.45 (0.18, 1.14)	0.092
Tumour category (T1/2 *versus* T3/4)	0.73 (0.41, 1.29)	0.276
Tumour size ≤ 2 cm	1.02 (0.55, 1.89)	0.957
Lymph node status ≥ N1	1.06 (0.70, 1.61)	0.784
Metastasis category M1	2.82 (1.72, 4.61)	< 0.001
Tumour grade (G1 *versus* G2/G3)	0.67 (0.40, 1.11)	0.119
Textbook outcome	0.60 (0.40, 0.89)	0.011

Values in parentheses are 95% confidence intervals.

## Discussion

In the present study of surgically resected pNETs, there was a high rate of TO, which was achieved in almost two-thirds of patients (64.1%). The TO rate was higher after organ-sparing resections (> 70%), underscoring the safety and quality of surgical treatment for pNETs, also for smaller tumours. Additionally, the institutional findings suggest that a TO is not only a quality benchmark but also functions as a prognostic factor for oncological outcomes, despite TO being a clinical and not a pathological endpoint.

The association between TO and pNETs from an oncological point of view was particularly interesting as the natural behaviour of these tumours differs fundamentally from that of PDAC, with strong implications for oncological outcomes^29^. In PDAC, it has already been shown that postoperative complications lead to impaired OS and higher rates of recurrent disease in the early phase of oncological surveillance; however, the effects on long-term outcomes may not be so obvious in a generally more aggressive cancer^30^. The more indolent nature of pNETs may allow a clearer assessment of perioperative complications and the potential association with recurrent disease and long-term survival during follow-up. It is well known that complications lead to systemic inflammation and impaired recovery, which increase the risk of recurrent disease in various cancer entities, but no data on this topic have been available for pNETs so far^31–34^. In the present cohort, a TO was associated with improved OS and, although DFS did not reach statistical significance in all subgroups, it was confirmed as an independent predictor in the multivariable analysis.

The literature on TO in pNETs remains limited, with few studies exploring its prognostic significance. Heidsma *et al*.^20^ analysed TO in a large multi-institutional cohort of 821 patients from the European Neuroendocrine Society database and reported a lower TO rate of 49.3%. However, the authors also found a significant correlation between TO and improved DFS. On the other hand, Partelli *et al*.^21^ focused exclusively on non-functioning pNETs undergoing pancreatoduodenectomy and did not find a difference in survival between patients with or without a TO (TO rate 32%). This was even more interesting as their TO definition also included a strict R0 resection margin as well as a minimum number of ≥ 12 harvested lymph nodes which were well known prognostic factors in pNETs and would, therefore, suggest an even stronger association with oncological outcomes. More recently, Chen *et al*.^35^ assessed the ‘ideal outcome’ (absence of in-hospital death, severe complications, POPF, reoperation, hospital stay > 75th percentile, and readmission) and observed lower ideal outcome rates for pNET compared with PDAC, mainly owing to higher rates of POPF (32.1 *versus* 7.9%). In contrast, the present cohort had a lower POPF rate, of 21% after pancreatoduodenectomy, which may explain the higher TO rate and a clearer link to long-term survival^36^.

One of the main challenges when comparing TO across these studies is the lack of a consistent definition. Some studies included pathological parameters, whereas others focused only on clinical outcomes^12,16,19,37,38^. In the present study, a clinical definition of TO was used without any histopathological criteria^15^. This allows broad application—also including organ-sparing resections—and avoids bias, for example in definition of R0 resections^39^. In addition, by excluding factors such as length of hospital stay as incorporated in the ‘ideal outcome’, TO allows better comparability across different healthcare systems and institutional policies^40^. Altogether, the definition of TO used in this study covers a relevant portion of patients with pNETs and allows its effect on oncological outcomes to be determined even without direct pathological surrogate markers.

This study confirmed several risk factors for failure to achieve TO, such as older age, raised BMI, higher ASA grade, advanced tumour stage, and longer operating time, which were also known from other diseases in which a TO was applied^41–43^. Interestingly, minimally invasive surgery did not correlate with higher TO rates, a finding that warrants further investigation as minimally invasive approaches become increasingly common^44^. One reason might be the selection bias for these cases or the learning curve in the early phase of implementation of minimally invasive procedures^45^. Importantly, current TO systems might not account for benefits associated with minimally invasive surgery, such as reduced pain or faster recovery^46,47^.

This study had several limitations: its retrospective design, the inclusion of different resection types including enucleations and segmental resections, and the inclusion of total pancreatectomies that might have influenced the incidence of POPF, although the proportion of this procedure in the entire cohort was very small. Different disease stages as well as functioning and non-functioning pNETs were included, which may affect the generalization of the data to more restrictive studies. Finally, as the study was conducted in a single high-volume centre, the extent to which these findings can be generalized to institutions with lower surgical caseloads remains uncertain.

In conclusion, the present findings have confirmed TO as an important quality metric in pancreatic surgery for pNETs. Besides perioperative quality metrics, TO correlated with long-term oncological outcomes, highlighting the interaction between surgical quality and cancer biology. Improving TO rates may not only improve short-term outcomes but also affect recurrence and long-term survival. Future studies should include prospective trials that stratify patients according to achievement of TO and assess whether closer postoperative follow-up of those failing to achieve a TO may improve long-term outcomes.

## Supplementary Material

zraf143_Supplementary_Data

## Data Availability

Study data are available on request.
