# Supplementary material for: Textbook outcome following surgery for pancreatic neuroendocrine tumours: retrospective study
Source: BJS Open. 2025 Dec 1;9(6):zraf143. doi: 10.1093/bjsopen/zraf143 (PMC12667269; doi:10.1093/bjsopen/zraf143)
Supplement: zraf143_Supplementary_Data [file zraf143_supplementary_data.docx]

**Textbook Outcome Following Surgery for Pancreatic Neuroendocrine Tumors**

Fabiola A. Bechtiger, Zoltan Czigany, Magdalena Lewosinska, Benedict Kinny-Köster, Max Heckler, Ingmar F. Rompen, Niels Siegel, Viola Pleines, Maximilian Kryschi, Jörg Kaiser, Mohammed Al-Saeedi, Christoph W. Michalski, Markus W. Büchler, Martin Loos, Thomas Hank

Department of General, Visceral and Transplant Surgery, Heidelberg University Hospital, Germany

**Corresponding author.** Thomas Hank, MD Department of General, Visceral and Transplant Surgery

Heidelberg University Hospital, Im Neuenheimer Feld 420, 69120 Heidelberg, Germany

Thomas.hank@med.uni-heidelberg.de

## ORCID ID; 0000-0001-8087-2295

**Supplementary Materials - Index**

| **Supplementary Figures and Tables** |  |
| --- | --- |
| Supplement Figure 1a: Disease-free survival of patients undergoing formal resection for pNEN, excluding patients that died within 30 days after surgery, n=456  Five-year disease-free survival with TO 67.4%, without TO 61.8%, p=0.096 | *pag. 2* |
| Supplement Figure 1b: Overall survival of patients undergoing formal resection for pNEN, excludinig patients that died within 30 days after surgery, n=456  Five-year overall survival with TO 83.9%, without TO 74.8%, p=0.012  median survival not achieved in both groups | *pag. 2* |

**Supplementary Figures**

**S1: Disease-free and overall survival of patients undergoing formal resections for pNEN, n=456** Excluding patients that died within 30 days after surgery

A B

**
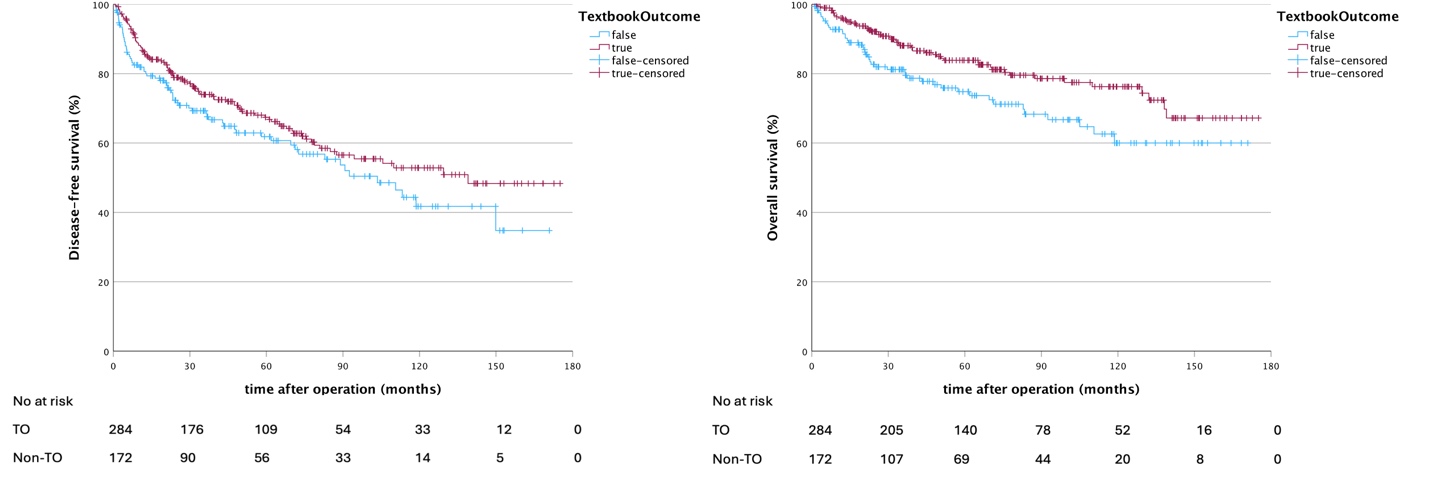
**

Supplement Figure 1A: Five-year disease free survival with TO 67.4%, without TO 61.8%, p=0.096
median survival with TO 139 months, without TO 103.5 months (CI 95% 75.4-131.5 months)

Supplement Figure 1B: Five-year overall survival with TO 83.9%, without TO 74.8%, p=0.012
median survival not achieved in both groups
